# Oxidative stress level is not associated with survival in terminally ill cancer patients: a preliminary study

**DOI:** 10.1186/1472-684X-13-14

**Published:** 2014-03-21

**Authors:** Chang Hwan Yeom, Youn Seon Choi, Hong Yup Ahn, Su Hey Lee, In Cheol Hwang

**Affiliations:** 1 Ucell clinic, Seoul, Republic of Korea; 2 Department of Family Medicine, Korea University Guro Hospital, Seoul, Republic of Korea; 3 Department of Statistics, Dongguk University, Seoul, Republic of Korea; 4 Palliative Care Unit, Division of Cancer Control & Prevention, Incheon Regional Cancer Center, 1198 Guwol-dong, Namdong-gu, Incheon 405-760, Republic of Korea

## Abstract

**Background:**

While cancer patients have higher oxidative stress (OS) and lower antioxidant activity, evidence for the association of these parameters with survival in patients with terminally ill cancer is lacking.

**Methods:**

We followed 65 terminal cancer patients prospectively. We assessed their performance status, some symptoms, and serum levels of vitamin C and OS level. The Gehan’s generalized Wilcoxon test was used to examine the association between survival times and variables.

**Results:**

Subjects’ performance status was very poor and they had a high level of OS and a low level of vitamin C. No significant association of these two parameters with survival time was noted (p-value, 0.637 for high OS and 0.240 for low vitamin C). Poor performance status was independently related to high OS status after adjusting for potential confounders (adjusted OR, 4.45; p-value, 0.031).

**Conclusions:**

In this study, OS was not associated with survival of terminally ill cancer patients and its prognostic role requires further study.

## Background

Prognostication of life expectancy is a significant clinical commitment for clinicians involved in oncology, especially in hospice-palliative care settings. Accurate estimates provide patients and families with a time frame in which to emotionally and financially prepare for the death [1]. However, studies on survival prediction in terminally ill patients suggest that it is very difficult to make an accurate prognosis and even experienced palliative care physicians find such a prediction difficult [2].

Patients with malignant tumors have higher oxidative stress (OS) by tumor growth itself and/or increased systemic inflammatory response [3,4]. OS is defined as a state in which the level of toxic reactive oxygen intermediates overcomes the endogenous antioxidant defenses. OS can therefore result from either excess production of free radicals or depletion of antioxidant defenses [5]. Meanwhile, vitamin C is a well-known antioxidant that humans are unable to synthesize and must obtain from an exogenous source. Reductions in vitamin C intake are associated with illness, hospitalization, and institutionalization. A population-based cohort study with breast cancer patients demonstrated that women consuming the highest tertile of vitamin C were significantly more likely to survive compared to those in the lowest tertile of intake [6]. Furthermore, a recent study reported that patients with low plasma levels of vitamin C have a significantly worse prognosis than patients with normal levels [7].

While a few studies have shown a correlation between survival and either serum vitamin C level or OS in cancer patients, direct evidence for this association in terminally ill cancer patients is lacking. Therefore, we aimed primarily to determine the relationship between survival and serum vitamin C or OS level in terminally-ill cancer patients. In addition, even if not associated with survival, we investigated whether these specific laboratory findings were connected with performance status (PS).

## Methods

### Design and subjects

**Figure 1:**
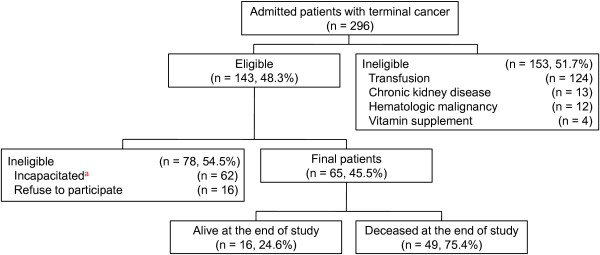
**Flowchart of patient recruitment. **^a^physician-assessed.

This prospective observational study was carried out at two hospice-palliative care units. From January 2012 to June 2012, we identified 296 consecutive terminally ill cancer patients who were admitted to facilities. A terminal cancer patient was defined as someone with progressive advanced cancer for whom conventional anticancer therapy was no longer indicated [1]. No subject included in the study had taken a vitamin supplement during the two days before enrollment, had received a blood transfusion or albumin during the one week before enrollment, had a chronic kidney disease, or had a hematologic malignancy. In addition, physicians determined eligibility for participation based on their capacity to communicate and to understand the aim of the study. Data of 65 patients were analyzed finally and a flowchart of patient recruitment depicted in Figure 1. This study was conducted in accordance with the Declaration of Helsinki. Written informed consent was obtained from each subject. The study was approved by Local Research Ethical Committee of Gachon University Gil Hospital (GIRBA2614) and the Korea University Guro Hospital (MD11033).

### Clinical examination

Data on demographics and clinical information were collected by an experienced palliative care team of physicians and registered nurses: included information were age, sex, cancer site, Eastern Cooperative Oncology Group (ECOG) performance status, palliative prognostic score [8], presence of fever and anorexia, and evidence of infection. The ECOG performance status is an observer-rated scale of patient physical ability using numbers ranging from 0 (able to carry out all normal activities) to 4 (completely disabled) [9]. Anorexia was defined as less than five spoons per meal (about one-third amount of routine meal). Patients also provided clinical information on average pain score by numerical rating scale and presence of some symptoms (dysphagia and dyspnea on exertion). Dysphagia and dyspnea were determined by the following yes-no question: “In the past 24 h, have you been difficult to swallow/short of breath on exertion?” Survival times were measured from the date of enrollment in the study.

### Blood assays

In all subjects, blood samples were obtained from venipuncture of an antecubital vein. Blood samples were used to measure complete blood cell count (white blood cell and lymphocyte proportion), liver function (albumin and total bilirubin), uric acid, creatinine, lactate dehydrogenase (LDH) and C-reactive protein (CRP). We determined oxidative stress levels by the Free Oxygen Radical Test (FORT; Free Oxygen Radicals Monitor Plus, Callegari, Italy), a colorimetric assay based on the ability of transition metals, such as iron, to catalyze the breakdown of hydroperoxides (ROOH) into derivative radicals, according to Fenton’s reaction. Results are expressed as FORT units (U.F.), where 1 U.F. corresponds to 0.26 mg/l H_2_O_2_. The assay is completed in 6 minutes. Data suggest that FORT can satisfactorily assess the level of oxidative radicals in whole blood [10]. We also measured serum vitamin C concentration by High Performance Liquid Chromatography, which has been validated as a rapid an specific measurement of vitamin C [11].

### Statistical analysis

Data are presented as median (interquartile range) or number (%). We dichotomized variables and chose a cut-off point at the reference range or median values. To examine the association between survival times and variables, and we used Gehan’s generalized Wilcoxon test. We evaluated the associations between OS and PS using the logistic regression model. We used SPSS statistical package version 16.02 (SPSS, Chicago, IL, USA). *P* values less than 0.05 were considered significant.

## Results

### Patient population and clinical characteristics

The demographic and clinical characteristics of the patients included in this study are shown in Table 1. In brief, most of the patients were between 58 and 76 years old and there were similar proportions by gender (male, 52.3%). Nearly half of the patients had lung or gastrointestinal tract cancer (44.6%). As would be expected in hospice-palliative care units, the PS of the subjects was poor: 96.9% of patients were ECOG 3 or 4. Median survival was 19 days (95% CI, 11–32 days). The median values of LDH, albumin, vitamin C and OS were outside their reference range.

**Table 1:** Characteristics of study patients (N = 65)

	**n (%)**
Age^a^, years	66 (58–76)
Survival time^b^, days	19 (11–32)
Sex	
Male	34 (52.3)
Female	31 (47.7)
Primary cancer site	
Lung	16 (24.6)
Gastrointestinal	13 (20.0)
Pancreas	11 (16.9)
Hepatobiliary	10 (15.4)
Others	15 (23.1)
ECOG performance status	
2-3	44 (67.7)
4	21 (32.3)
Symptom presence	
Fever	24 (36.9)
Current infection	29 (44.6)
Anorexia	59 (90.8)
Dysphagia	24 (36.9)
Dyspnea on exertion	15 (23.1)
Uncontrolled pain (NRS ≥ 4)	31 (47.7)
Palliative prognostic score	
0-5.5	25 (38.5)
5.6-11.0	24 (36.9)
11.1-17.5	16 (24.6)
Laboratory findings^a^ (reference range)	
Bilirubin, mg/dL (0.2-1.2)	0.6 (0.3-1.2)
Creatinine, mg/dL (0.5-1.2)	0.6 (0.4-0.9)
Lactate dehydrogenase, U/L (200–485)	557 (427–776)
Uric acid, mg/dL (2.5-8.3)	3.7 (2.6-6.0)
Albumin, g/dL (3.5-5.2)	3.0 (2.6-3.3)
C-reactive protein, mg/dL (< 0.5)	16.3 (4.0-74.5)
Vitamin C, μg/mL (1.9-15.0)	0.44 (< 0.03-0.89)
Oxidative stress^c^, U.F. (160–310)	412.9 (305.1-539.2)

*Abbreviations:* ECOG Eastern Cooperative Oncology Group, NRS numerical rating score.

^a^Expressed as median (interquartile range).

^b^Expressed as median (95% CI).

^c^By Free Oxygen Radical Test.

### Prognostic factors on survival

In univariate survival analysis, male gender (HR, 1.95; *P* = 0.024), poor PS (HR, 1.91; *P* = 0.032), higher palliative prognostic score (HR, 2.30; *P* < 0.01), and elevated serum creatinine levels (HR, 4.22; *P* < 0.01) were significant predictors of decreased survival time (Table 2). No significant influences of OS or vitamin C level on survival time were noted (*P* = 0.24 for vitamin C and *P* = 0.637 for OS, respectively).

**Table 2:** Patient characteristics and univariate survival analysis

	**No. of patients**	**MST (days)**	**HR (95% CI)**	***P *** **Value**^**b**^
Age (years)				
	≤ 65	32	23	1	
	> 65	33	11	1.19 (0.68-2.09)	0.544
Sex					
	Female	31	31	1	
	Male	34	11	1.95 (1.09-3.50)	0.024
Primary tumor site (any type)					
	Lung	16	32	0.71 (0.37-1.36)	0.300
	Gastrointestinal	13	14	1.34 (0.68-2.66)	0.401
	Pancreas	11	17	1.38 (0.67-2.86)	0.387
	Hepatobiliary	10	10	1.39 (0.65-3.00)	0.396
	Others	15	19	0.70 (0.34-1.45)	0.332
ECOG PS					
	2-3	44	23	1	
	4	21	10	1.91 (1.06-3.45)	0.032
Palliative prognostic score					
	< 7.5^a^	31	22	1	
	≥ 7.5^a^	34	10	2.30 (1.27-4.17)	< 0.01
Laboratory variables					
	Bilirubin (mg/dL)				
WNR	47	22	1		
Hyperbilirubinemia	18	11	1.45 (0.79-2.69)	0.233	
	Creatinine (mg/dL)				
WNR	56	22	1		
Elevated	9	6	4.22 (1.98-9.02)	< 0.01	
	Lactate dehydrogenase (U/L)				
WNR	19	31	1		
Elevated	41	15	1.25 (0.65-2.42)	0.500	
	Uric acid (mg/dL)				
WNR	54	22	1		
Hyperuricemia	8	12	2.10 (0.92-4.82)	0.078	
	Albumin (g/dL)				
WNR	11	31	1		
Hypoalbuminemia	54	17	1.52 (0.68-3.39)	0.311	
	C-reactive protein (mg/dL)				
< 16.3^a^	33	18	1		
≥ 16.3^a^	32	19	0.60 (0.33-1.07)	0.084	
	Serum vitamin C (μg/mL)				
≥ 0.44^a^	33	17	1		
< 0.44^a^	32	20	0.71 (0.40-1.26)	0.240	
	Oxidative stress (U.F.)				
< 412.9^a^	32	22	1		
≥ 412.9^a^	33	15	1.15 (0.65-2.02)	0.637	

*Abbreviations:* ECOG PS Eastern Cooperative Oncology Group Performance Status, WNR within normal range, MST median survival time, HR hazard ratio, CI confidence interval.

^a^Median value.

^b^Gehan’s generalized Wilcoxon method.

### OS and PS

Table 3 lists the associated factors with high OS level. Subjects with poor PS (ECOG = 4) had significantly higher proportion with high OS level (OR, 3.61; *P* = 0.025). This relationship remained robust after adjusting for potential confounders, such as age group, sex, presence of fever and hypoalbuminemia (OR, 4.45; *P* = 0.031).

**Table 3:** Oxidative stress status and baseline clinical characteristics

	**Higher oxidative stress (≥ median, 412.9 U.F.)**		
	**OR**	**95% CI**	***P *** **Value**
Aged (> 65 yrs)	1.06	0.40-2.82	0.914
Sex (male)	1.98	0.74-5.31	0.176
Palliative prognostic score (≥ 7.5^a^)	1.20	0.45-3.18	0.714
Symptoms (presence)			
Fever	3.79	1.29-11.18	0.016
Current infection	1.38	0.52-3.67	0.524
Anorexia	0.48	0.08-2.84	0.421
Dysphagia	2.13	0.76-2.97	0.151
Dyspnea on exertion	1.63	0.50-5.25	0.417
Uncontrolled pain (NRS ≥ 4)	1.75	0.66-4.69	0.263
Laboratory findings			
Hyperbilirubinemia	0.70	0.24-2.10	0.529
Elevated creatinine	1.25	0.30-5.15	0.757
Elevated lactate dehydrogenase	1.64	0.56-4.82	0.371
Hyperuricemia	0.77	0.21-2.84	0.699
Hypoalbuminemia	0.18	0.04-0.90	0.037
High^a^ C-reactive protein	1.54	0.58-4.10	0.385
Low^a^ vitamin C	0.74	0.28-1.95	0.537
Performance (ECOG = 4)			
Unadjusted	3.61	1.18-11.08	0.025
Adjusted^b^	4.45	1.14-17.33	0.031

*Abbreviations:* NRS numerical rating score, ECOG Eastern Cooperative Oncology Group.

^a^Based on the median value.

^b^Adjusted for age group, sex, fever, and hypoalbuminemia.

## Discussion

The idea of being able to prognosticate from the results of a simple blood test is very appealing to palliative care physicians. The need for a blood sample must also be weighed against the likely clinical advantage for the individual patient. Biologic parameters have not been as widely investigated as clinical parameters in terminally ill cancer patients [12], and a more accurate evaluation of these variables in relation to prognosis is undoubtedly warranted.

To our knowledge, this is a rare study that investigated the clinical effectiveness of measuring serum vitamin C or OS in terminally ill cancer patients. In this study, the main findings were that: (i) these parameters had no prognostic significance; (ii) serum OS level was associated with PS of patients; and (iii) serum vitamin C level was not correlated with OS level.

It is well known that cancer patients are characterized by higher levels of OS markers and lower levels of serum vitamin C than healthy controls; there is a scarcity of literature addressing the association with survival of advanced cancer patients at the end of life [13]. Mayland et al. [7] reported that low plasma vitamin C concentrations were associated with shorter survival in terminally ill cancer patients. But those findings were not confirmed by multivariate analysis and they did not address the level of OS, which could be a predictor of cancer-specific survival [14]. In the current study, we were unable to demonstrate prognostic significance of serum vitamin C or OS on survival. The lack of significance could be due to (i) true negative finding: these parameters are not helpful to discriminate the patients with a short survival, or (ii) false negative findings: small sample size or artificial cutoff value.

While several studies have shown that OS level was associated with the cancer patient’s PS [15,16], little work has been done in terminally ill cancer patients. The distressing symptoms experienced by cancer patients may be related to OS: these symptoms may also lead to the patients’ poor PS [17]. Especially in the terminal stage, deranged energy metabolism, which may account for symptoms such as nausea/vomiting, anorexia and cachexia that cause malnutrition, could accelerate the production of free radicals. Additionally, a previous study reported that the OS levels decreased significantly after antioxidant treatment regardless of patient’s PS [15], although these reductions might not lead to performance improvement [17,18]. Further well-controlled clinical trials are needed to clarify the clinical effect of intervention.

Based on the complex situation caused by an imbalance between OS and antioxidant ability, it is plausible that separate assessments of OS and antioxidant status do not provide sufficient information [19]. For that reason we assessed both parameters, but could not find any correlation between them. With healthy adults, Block et al. [20] found that serum vitamin C was inversely correlated with biomarkers of OS even after adjusting for other antioxidants. The antioxidant system counteracts OS constantly and prevents certain damage by scavenging reactive oxygen species [21], but this balance may be limited to healthy subjects. As the disease progresses and death nears, OS levels escalate markedly without linear reduction of antioxidant capacity. Therefore, a higher OS level may have more influence on the outcome for terminal cancer patients than decreased antioxidant biomarkers. Two recent studies by Vera-Ramirez et al. with breast cancer patients back this up. In non-metastatic breast cancer patients, they demonstrated that both antioxidant capacity and OS biomarkers had significant association with survival rate [22]. In contrast, in a metastatic setting with the same protocol, only OS parameters significantly influenced the survival rates of the patients [14].

The current study contains some limitations that should be acknowledged. First, this sample of patients is too small. Large-scale studies are needed to confirm our findings, because short survival time is unavoidable when dealing with subjects in a hospice setting. Second, by using a single measurement, we have likely underestimated the association of serum vitamin C level and OS status with survival. Third, we only adjusted for confounders statistically and could not exclude acute inflammatory conditions or anti-inflammatory medication users, which can affect OS [23]. However, terminally ill cancer patients are very susceptible to infection [24], and they have also taken multiple drugs due to a wide variety of symptoms and associated comorbid conditions [25]. Finally, some symptoms such as anorexia, dysphagia and dyspea on exertion were not evaluated by a validated symptom assessment tool.

## Conclusions

Our study demonstrates that OS status was not associated with survival of cancer patients at the very end of life. Further studies are needed in order to elucidate the relationship between survival and OS.

## Competing interests

CHY is the chairperson of the Korean Association for Vitamin Research. All authors have no potential conflicts of interest concerning this article.

## Authors’ contributions

CHY, YSC, and ICH designed study. CHY, YSC and ICH wrote the manuscript. YSC, SHL and ICH conducted study and collected the data. AHY and ICH analysed the data. CHY, YSC and ICH interpreted the data. All authors read and approved the final manuscript.

## Pre-publication history

The pre-publication history for this paper can be accessed here:

http://www.biomedcentral.com/1472-684X/13/14/prepub
